# Fabrication of a new type of organic-inorganic hybrid superlattice films combined with titanium oxide and polydiacetylene

**DOI:** 10.1186/1556-276X-7-71

**Published:** 2012-01-05

**Authors:** Kwan-Hyuck Yoon, Kyu-Seok Han, Myung-Mo Sung

**Affiliations:** 1 Department of Chemistry, Hanyang University, Seoul, 133-791, South Korea

**Keywords:** organic-inorganic nanohybrid superlattices, molecular layer deposition, atomic layer deposition, polydiacetylene.

## Abstract

We fabricated a new organic-inorganic hybrid superlattice film using molecular layer deposition [MLD] combined with atomic layer deposition [ALD]. In the molecular layer deposition process, polydiacetylene [PDA] layers were grown by repeated sequential adsorption of titanium tetrachloride and 2,4-hexadiyne-1,6-diol with ultraviolet polymerization under a substrate temperature of 100°C. Titanium oxide [TiO_2_] inorganic layers were deposited at the same temperatures with alternating surface-saturating reactions of titanium tetrachloride and water. Ellipsometry analysis showed a self-limiting surface reaction process and linear growth of the nanohybrid films. The transmission electron microscopy analysis of the titanium oxide cross-linked polydiacetylene [TiOPDA]-TiO_2 _thin films confirmed the MLD growth rate and showed that the films are amorphous superlattices. Composition and polymerization of the films were confirmed by infrared spectroscopy. The TiOPDA-TiO_2 _nanohybrid superlattice films exhibited good thermal and mechanical stabilities.

**PACS: **81.07.Pr, organic-inorganic hybrid nanostructures; 82.35.-x, polymerization; 81.15.-z, film deposition; 81.15.Gh, chemical vapor deposition (including plasma enhanced CVD, MOCVD, ALD, etc.).

## Background

Organic-inorganic hybrid superlattice films have an attractive potential for the creation of new types of functional materials by combining organic and inorganic properties. The hybrid superlattice films provide both the stable and distinguished optical or electrical properties of inorganic constituents and the structural flexibility of organic constituents. Furthermore, such hybrid superlattice films show unique optical and electrical properties which differ from their constituents [1-3]. They provide the opportunity for developing new materials with synergic effects, leading to improved performance or useful properties. A key factor to utilize organic-inorganic hybrid films is the ability to prepare high quality multilayers in the simplest and most reliable method. The ability to assemble one monolayer of hybrid films at a time provides control over thickness, composition, and physical properties with a single-layer precision. Such monolayer control provides an important path for the creation of new hybrid materials for organic-inorganic electronic devices and molecular electronics.

Recently, we developed two-dimensional polydiacetylene [PDA] with hybrid organic-inorganic structures using molecular layer deposition [MLD] [4]. MLD is a gas-phase layer-by-layer growth process, analogous to atomic layer deposition [ALD] that relies on sequential, self-limiting surface reactions [5-13]. In the MLD method, the high-quality organic PDA thin films can be quickly formed with monolayer precision under ALD conditions (pressure, temperature, etc.). The MLD method can be combined with ALD to take advantages of the possibility of obtaining organic-inorganic hybrid thin films. The advantages of the MLD technique combined with ALD include accurate control of film thickness, good reproducibility, large-scale uniformity, multilayer processing ability, and excellent film qualities. Therefore, the MLD method with ALD [MLD-ALD] is an ideal fabrication technique for various organic-inorganic nanohybrid thin films.

Herein, we report a fabrication of titanium oxide cross-linked polydiacetylene [TiOPDA]-titanium oxide [TiO_2_] organic-inorganic nanohybrid thin films using the MLD-ALD method. In this MLD process, the PDA organic layers were grown by repeated sequential ligand-exchange reactions of titanium tetrachloride [TiCl_4_] and 2,4-hexadiyn-1,6-diol [HDD] with UV polymerization. The TiO_2 _inorganic nanolayers were prepared by ALD using TiCl_4 _and water. The prepared TiOPDA-TiO_2 _nanohybrid thin films exhibited good thermal and mechanical stability.

## Experimental details

### Preparation of Si substrates

The Si (100) substrates used in this research were cut from p-type (100) wafers with a resistivity in the range of 1 to 10 Ω cm. The Si substrates were initially treated by a chemical cleaning process proposed by Ishizaka and Shiraki which involved degreasing, HNO_3 _boiling, NH_4_OH boiling (alkali treatment), HCl boiling (acid treatment), rinsing in deionized water, and blow-drying with nitrogen to remove contaminants and grow a thin protective oxide layer on the surface [14].

### Atomic layer deposition of TiO_2 _thin film

The oxidized Si (100) substrates were introduced into the ALD system Cyclic 4000 (Genitech, Daejon, Korea). The TiO_2 _thin films were deposited onto the substrates using TiCl_4 _(99%; Sigma-Aldrich Corporation, St. Louis, MO, USA) and water as ALD precursors [14]. Ar served as both a carrier and a purging gas. The TiCl_4 _and water were evaporated at 30°C and 20°C, respectively. The cycle consisted of a 1-s exposure to TiCl_4_, 5-s Ar purge, 1-s exposure to water, and 5-s Ar purge. The vapor pressure of the Ar in the reactor was maintained at 100 mTorr. The TiO_2 _thin films were grown at 100°C under a pressure of 100 mTorr.

### Molecular layer deposition

TiOPDA thin films were deposited onto the Si substrates using TiCl_4 _and HDD (99%; Sigma-Aldrich Corporation, St. Louis, MO, USA) in the MLD chamber. Ar served as both a carrier and a purging gas. TiCl_4 _and HDD were evaporated at 30°C and 80°C, respectively. The cycle consisted of a 1-s exposure to TiCl_4_, 5-s Ar purge, 10-s exposure to HDD, and 50-s Ar purge. The vapor pressure of the Ar in the reactor was maintained at 100 mTorr. The deposited HDD layer was exposed to UV (254 nm, 100 W) for 30 s. The TiOPDA thin films were grown at 100°C under a pressure of 100 mTorr.

### Sample characterization

The thicknesses of the thin films were evaluated using an ellipsometer (AutoEL-II, Rudolph Research Analytical, Hackettstown, NJ, USA). UV-Visible [Vis] and Fourier transform infrared [FTIR] spectra were obtained using a UV-Vis spectrometer (Agilent 8453 UV-Vis, Agilent Technologies Inc., Santa Clara, CA, USA) and an FTIR spectrometer (FTLA 2000, ABB Bomem, Quebec, Quebec, Canada), respectively. All X-ray photoelectron [XP] spectra were recorded on a Thermo VG Sigma Probe spectrometer (FEI Co., Hillsboro, OR, USA) using Al Kα source run at 15 kV and 10 mA. The binding energy scale was calibrated to 284.5 eV for the main C 1s peak. Each sample was analyzed at a 90° angle relative to the electron analyzer. The samples were analyzed by a JEOL-2100F transmission electron microscope (JEOL Ltd., Akishima, Tokyo, Japan). Specimens for cross-sectional transmission electron microscopy [TEM] studies were prepared by mechanical grinding and polishing (approximately 10-μm thick) followed by Ar-ion milling using a Gatan Precision Ion Polishing System (PIPS™ Model 691, Gatan, Inc., Pleasanton, CA, USA).

## Results

**Figure 1:**
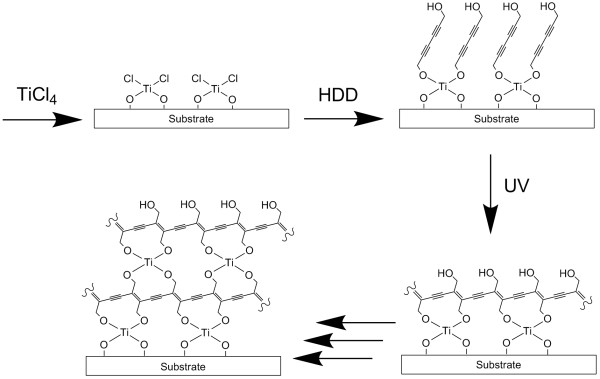
**Schematic outline**. Schematic outline of the procedure to fabricate TiOPDA films using molecular layer deposition.

Figure 1 shows a schematic outline for the present layer-by-layer synthesis of the TiOPDA films. First, the TiCl_4 _molecule was chemisorbed on substrate surfaces rich in hydroxyl groups via ligand exchange reaction to form the Cl-Ti-O species. Second, the Cl group of the chemisorbed titanium chloride molecule on the substrates was replaced by an OH group of HDD with the living HCl to form a diacetylene layer. The OH group of the diacetylene layer provides an active site for exchange reaction of the next TiCl_4_. Third, the diacetylene molecules were polymerized by UV irradiation to form a polydiacetylene layer. The TiOPDA thin films were grown under vacuum by repeated sequential adsorptions of TiCl_4 _and HDD with UV polymerization. The expected monolayer thickness for the ideal model structure of TiOPDA is about 6 Å.

**Figure 2:**
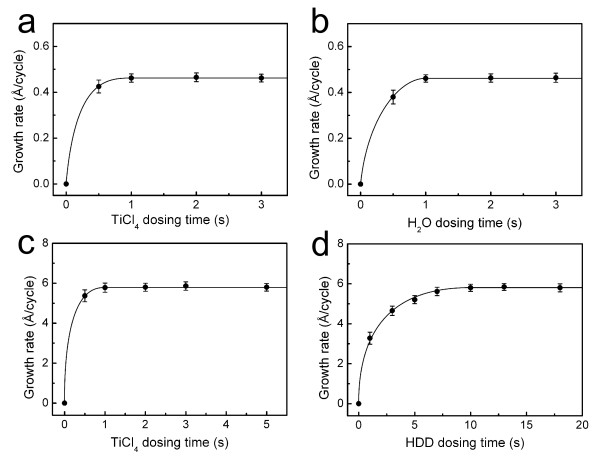
**Self-terminating growth graphs**. (**a**) Growth rate of TiO_2 _as a function of TiCl4 dosing time. (**b**) Growth rate of TiO2 as a function of H_2_O dosing time. (**c**) Growth rate of TiOPDA as a function of TiCl4 dosing time. (**d**) Growth rate of TiOPDA as a function of HDD dosing time.

TiO_2_-based organic-inorganic nanohybrid thin films were grown by MLD combined with ALD in the same deposition chamber. TiO_2 _inorganic nanolayers were grown by ALD using self-terminating surface reactions at 100°C, followed by deposition of the TiOPDA films using MLD; we name those organic-inorganic hybrid layers as TiOPDA-TiO_2_. To demonstrate that the surface reactions of the ALD and MLD processes are really self-limiting, the dosing times of the precursors were varied. Figure 2a, b shows that the TiO_2 _growth rate as a function of the TiCl_4 _and H_2_O dosing time is saturated when the pulse time exceeds 1 s, which indicates that the growth is self-limiting. In the MLD process, the TiOPDA growth rate as a function of the TiCl_4 _is saturated when the time exceeded 1 s, and the HDD dosing time is saturated when the time exceeded 10 s in Figure 2c, d. These saturation data indicate that the MLD growth is self-limiting. All the self-terminating growth experiments were performed in 100 cycles, and the measured growth rates for the ALD and MLD processes were about 0.46 and 6 Å per cycle, respectively.

**Figure 3:**
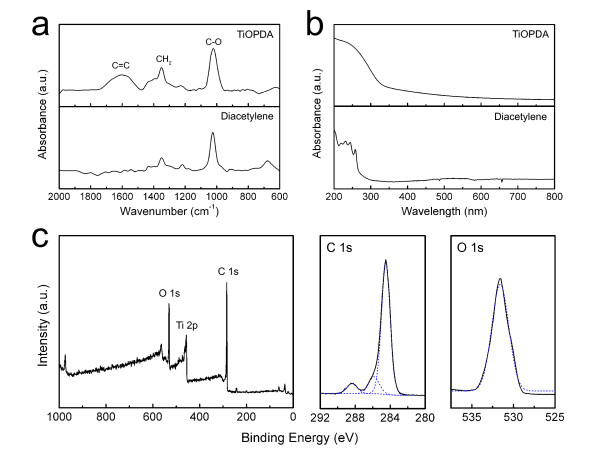
**Analysis data of TiOPDA films**. (**a**) FTIR spectra for the TiOPDA polymer and diacetylene films. (**b**) UV-Vis spectra for the TiOPDA polymer and diacetylene films. (**c**) XP survey and high resolution spectra for the TiOPDA polymer film.

To verify the formation of the TiOPDA polymer layer properly in the organic-inorganic superlattice film, the photopolymerization of the diacetylene organic layers was analyzed by FTIR spectroscopy. The TiOPDA films were deposited on KBr substrates by the MLD process in 1,000 cycles. Figure 3a illustrates IR spectra for the TiOPDA and diacetylene films. The prominent peak around 1,600 cm^-1 ^is due to C = C stretching, which confirms that diacetylene molecules in the films are polymerized by UV irradiation. The optical property of the TiOPDA film was investigated by UV-Vis spectroscopy. Figure 3b shows that the UV-Vis spectrum for the TiOPDA is similar to that of a conventional polydiacetylene [15]. The composition of the TiOPDA organic films was determined using XP spectroscopy. The survey and high resolution spectra of the TiOPDA films grown on a Si (100) substrate were shown in Figure 3c. The XP spectrum shows the photoelectron peaks for titanium, oxygen, and carbon. The ratio of peak area under titanium, oxygen, and carbon was 1:5.6:11.7 (Ti:O:C). The expected ratio from the ideal structure of TiOPDA is 1:4:12. The higher oxygen atomic percentage could be explained by the absorption of H_2_O into the TiOPDA [12]. The C 1s region in the high-resolution spectrum of the TiOPDA films can be deconvolved into three peaks. The C 1s peak at 284.5 eV is assigned to the conjugated carbons. The peaks at 286.0 and 288.4 eV are due to the carbons bound to the near electronegative oxygen [15,16].

**Figure 4:**
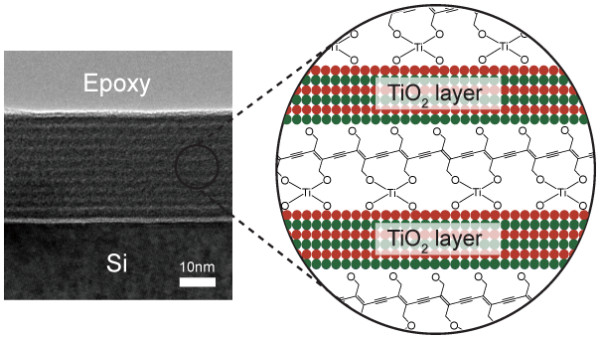
**TEM images**. TEM image of a typical TiOPDA-TiO_2 _nanohybrid thin film.

A typical TiOPDA-TiO_2 _nanohybrid thin film was grown on Si (100) substrates by repeating 50 cycles of ALD and 1 cycle of MLD in the same chamber at 100°C. The TEM image provides direct observation of the superlattice structure and confirms the expectation for the individual TiOPDA and TiO_2 _nanolayers in the hybrid thin film, as shown in Figure 4. The TiOPDA-TiO_2 _nanohybrid thin films were approximately 29-nm thick and consisted of ten [TiOPDA (0.6 nm)/TiO_2 _(2.3 nm)] bilayer subunits. The thermal stability of the TiOPDA-TiO_2 _films was studied by using TEM. The films were stable in air up to temperatures of about 400°C. This, together with the ability of the TiOPDA-TiO_2 _films to survive the TEM preparation process, indicates that they have good thermal and mechanical stability due to the titanium oxide crosslinkers of the polydiacetylene.

## Conclusions

We developed TiOPDA-TiO_2 _organic-inorganic hybrid superlattice films by MLD combined with ALD. In the MLD process, TiOPDA organic layers were grown under vacuum by repeated sequential adsorptions of 2,4-hexadiyne-1,6-diol and titanium tetrachloride with UV polymerization. In the ALD process, TiO_2 _inorganic nanolayers were deposited at the same chamber using alternating surface-saturating reactions of titanium chloride and water. The TiOPDA-TiO_2 _nanohybrid thin films that were prepared exhibit good thermal and mechanical stability, large-scale uniformity, and sharp interfaces.

## Competing interests

The authors declare that they have no competing interests.

## Authors' contributions

KHY performed the experiment, analyzed the data, and drafted the manuscript. KSH carried out TEM measurement. MMS conceived and designed the experiment. All authors read and approved the final manuscript.
